# Cow-hitch fixation in fracture hemiarthroplasty

**DOI:** 10.1016/j.jseint.2021.07.011

**Published:** 2021-09-11

**Authors:** Florian Grubhofer, Lukas Ernstbrunner, Elias Bachmann, Karl Wieser, Paul Borbas, Samy Bouaicha, Jon J.P. Warner, Christian Gerber

**Affiliations:** a Balgrist University Hospital, Department of Orthopedic Surgery, University of Zürich, Zürich, Switzerland; b Massachusetts General Hospital, Department of Orthopedic Surgery, Harvard Medical School, Boston, MA, USA

**Keywords:** Proximal humerus fracture, Hemiarthroplasty, Greater tuberosity reattachment, Lesser tuberosity reattachment, Cow Hitch Cerclage, Cadaver study

## Abstract

**Background:**

The treatment of complex proximal humerus fractures with hemiarthroplasty is associated with a high failure rate due to secondary displacement of the tuberosities. It was the aim of this in-vitro study to compare the mechanical stability of tuberosity reattachment obtained with the so-called “Cow-Hitch” (CH) cerclage compared with conventional tuberosity reattachment.

**Methods:**

A 4-part proximal humerus fracture was created in 10 fresh-frozen, human cadaveric shoulders. The greater and lesser tuberosity were reattached to the hemiarthroplasty stem with in total 4 CH Cerclages in the Cow-Hitch group. The conventional technique—recommended for the tested implant—was used in the control group using 6 sutures. A total of 5000 loading cycles with forces of 350N were applied, while motion (in mm) of the tuberosities was recorded in 3 directions (anteroposterior = AP, mediolateral = ML, inferosuperior = IS) with a telecentric camera.

**Results:**

After 5000 loading cycles, the CH group showed less fragment displacement (AP: 2.3 ± 2.3 mm, ML: 1.8 ± 0.9 mm, IS: 1.3 ± 0.5 mm) than the conventional group (AP: 9.8 ± 12.3 mm, ML: 5.5 ± 5.6 mm, IS: 4.5 ± 4.7 mm). The differences were not statistically significant (AP: *P* = .241; ML: *P* = .159; IS: *P* = .216). The lesser tuberosity fragment displacement in the CH group after 5000 cycles was less in the AP (2.3 ± 3.3 vs. 4.0 ± 2.8, *P* = .359) and IS (1.9 ± 1.2 vs. 3.1 ± 1.8; *P* = .189) directions but higher in the ML direction (7.2 ± 5.7 vs 6.3 ± 3.6, *P* = .963).

**Conclusions:**

In-vitro, “Cow-Hitch” cerclage results in mean greater tuberosity displacements of 2 mm and reliably prevents displacements greater than 5 mm. In contrast, the conventional fixation technique yields unreliable, variable stability with low to complete displacement upon cyclical loading.

Shoulder hemiarthroplasty (HA) is a recognized treatment method for complex proximal humeral fractures. While reverse total shoulder arthroplasty is preferred in elderly patients,^5^^,^^6^^,^^8^ HA is still considered for younger patients, if open reduction and internal fixation are not feasible.^16^

Even if good functional results after open reduction and internal fixation for head-split fractures and fracture dislocations were documented in few studies, the complication rate is even higher than that after HA.^3^^,^^15^, ^16^, ^17^ Nevertheless, the complication rate of HA is also not acceptable mainly because of secondary displacement with nonunion or malunion of mostly the greater but also the lesser turberosity.^2^^,^^9^^,^^14^^,^^16^

One reason for secondary tuberosity displacement is an unstable reattachment of the tuberosities. Accordingly, numerous tuberosity fixation techniques have been described, but the mechanical stability provided is unknown for most of them.^1^ In a previous biomechanical study, fixation of the greater tuberosity (GT) to a fracture reverse total shoulder arthroplasty stem using the Cow-Hitch (CH) cerclage was significantly more stable than fixation using the conventional (CON) fixation technique.^7^

Stable tuberosity reattachment is even more important in HA, and the question arises, whether a better fixation of the tuberosities to a HA fracture stem can be achieved using a CH technique.

The aim of this biomechanical study was to quantify the mechanical stability of fixation of the greater and lesser tuberosity (LT) comparing the CH with the technique recommended by the manufacturer of the used implant.

## Methods

After obtaining institutional review board approval, 12 fresh-frozen cadaveric human shoulders (Science Care, Phoenix, AZ, USA) were used for this study. To simulate the surgical condition as realistically as possible, the experiments were performed with muscle, ligaments, soft tissue, and skin left attached to the shoulders. The deep-frozen specimens were thawed over 12 hours in a 4°C refrigerator. The surgeries were performed by three different—fellowship-trained—shoulder surgeons (F.G., L.E., P.B.). The specimens were fixated with a spike gripper on the medial scapular margin and positioned in a simulated beach chair position. Through a deltopectoral approach, the proximal humerus was exposed. A four-part humerus fracture was created with a chisel. All fractures were computed tomography scanned. The fractures were grouped into 6 pairs according to the size of the greater and LT fragments which was measured in millimeters.

In addition, the bone density of all proximal humeri was determined according to the measurement method described by Rho et al, to guarantee comparability of the specimens.^13^ The head diameter was measured to define the implant head size, and the diameter of the humeral shaft was measured to plan the humeral stem size. The paired shoulders were then randomized to the fixation technique (CH vs. CON tuberosity fixation technique). The head fragment was removed. The greater and LT fragments were mobilized and grasped with MaxBraid sutures Nr. 5 (ZimmerBiomet, Warsaw, IN, USA) sutures. The fracture prosthesis (Anatomical Shoulder Fracture; Zimmer Biomet, Warsaw, In, USA,) was cemented (Refobacin Bone cement R; Zimmer Biomet) into the shaft with 20° of retroversion, the epicondylar axis of the distal humerus serving as reference. The height of the prosthetic stem was determined with the upper border of the pectoralis major tendon serving as a reference.^11^

### CON reattachment technique

**Figure 1:**
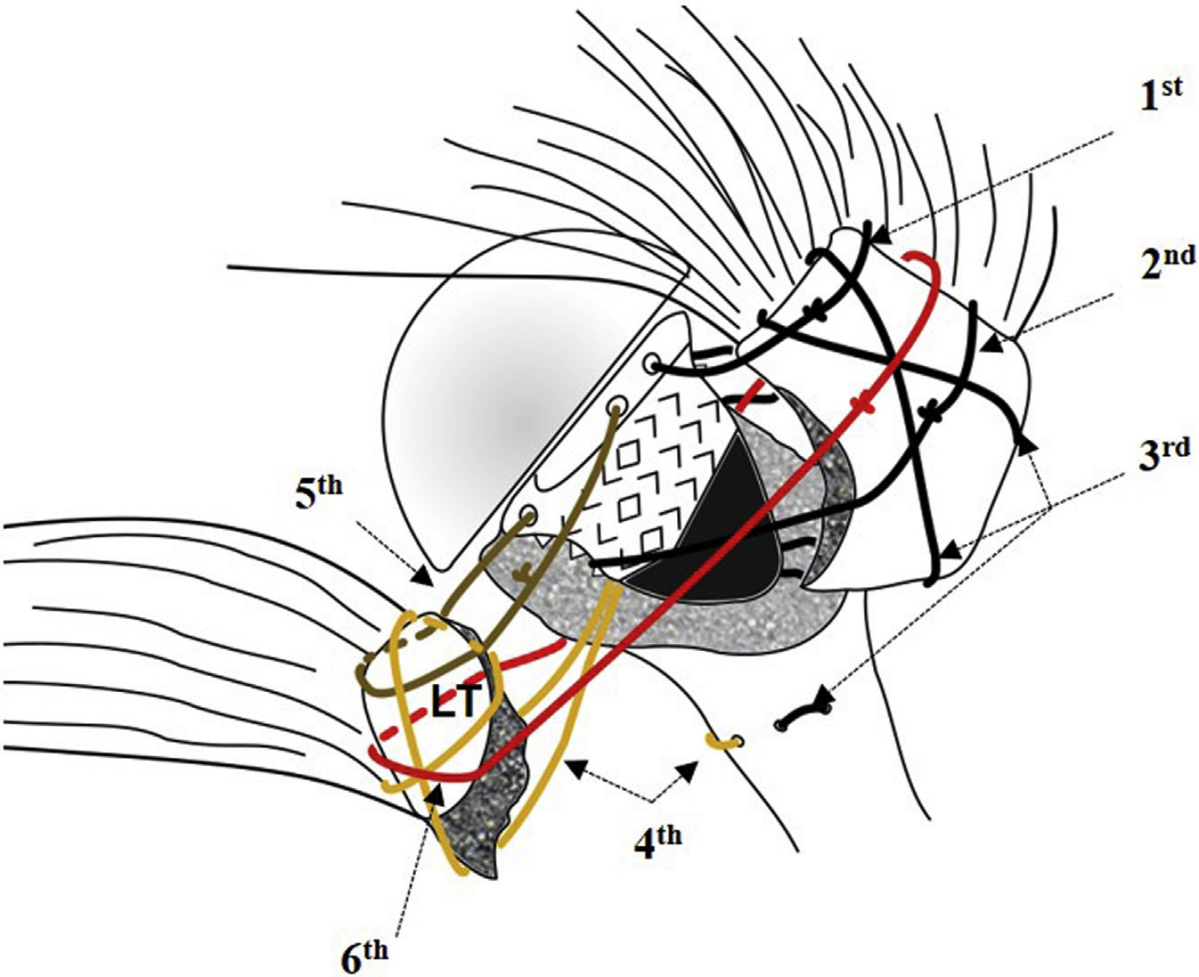
Reattachment of the greater tuberosity (GT) and the lesser tuberosity (LT) with the conventional technique using six (1^st^ to 6^th^) sutures. Find the exact description of each suture in the Methods section.

The CON technique was that recommended in the manual of the manufacturer (ZimmerBiomet Anatomical Shoulder Fracture System's Manual) (Fig. 1). In total, 6 MaxBraid sutures #5 (ZimmerBiomet) were used for the greater and LT reattachment. Three sutures were used for the GT and 2 for the lesser; 1 suture was passed around both tuberosities.

#### GT reattachment

The first suture was passed around the GT and through the lateral prosthetic shaft hole (Fig. 1, "1^st^"); the second suture was passed around the GT and through the medial prosthetic shaft hole (Fig. 1, "2^nd^"). The third suture was passed transosseously through the humerus first, and after implantation of the stem, the suture was passed in a vertical, figure-of-eight fashion around the GT (Fig. 1, "3^rd^"). All sutures were passed through the tendon-bone interphase of the GT and the supraspinatus and infraspinatus tendon. After anatomical reduction of the GT, the humerus was held in 30° of abduction and 20° of external rotation to tension and secure all sutures with 7 half hitches.

#### LT reattachment

The first suture was also passed transosseously before the shaft was implanted. After implantation, the suture was passed analog to the figure-of-eight suture of the GT around the LT (Fig. 1, "4^th^"). The second suture was passed around the LT and through the anterior hole of the prosthetic collar (Fig. 1, "5th"). The third suture (the so-called “around the world suture”) was passed around the LT, through the medial calcar hole around the (Fig. 1, "6^th^"). Again, all sutures were passed at the tendon-bone interphase between LT and subscapularis tendon. After anatomic reduction of the LT, the humerus was held in 20° of internal rotation, and the knots were tensioned with the most adjustable tension and secured with 7 half hitches. To prevent loosening of tension on the suture, a hemostat was used to secure the tension after setting the first half hitch.

### CH technique

**Figure 2:**
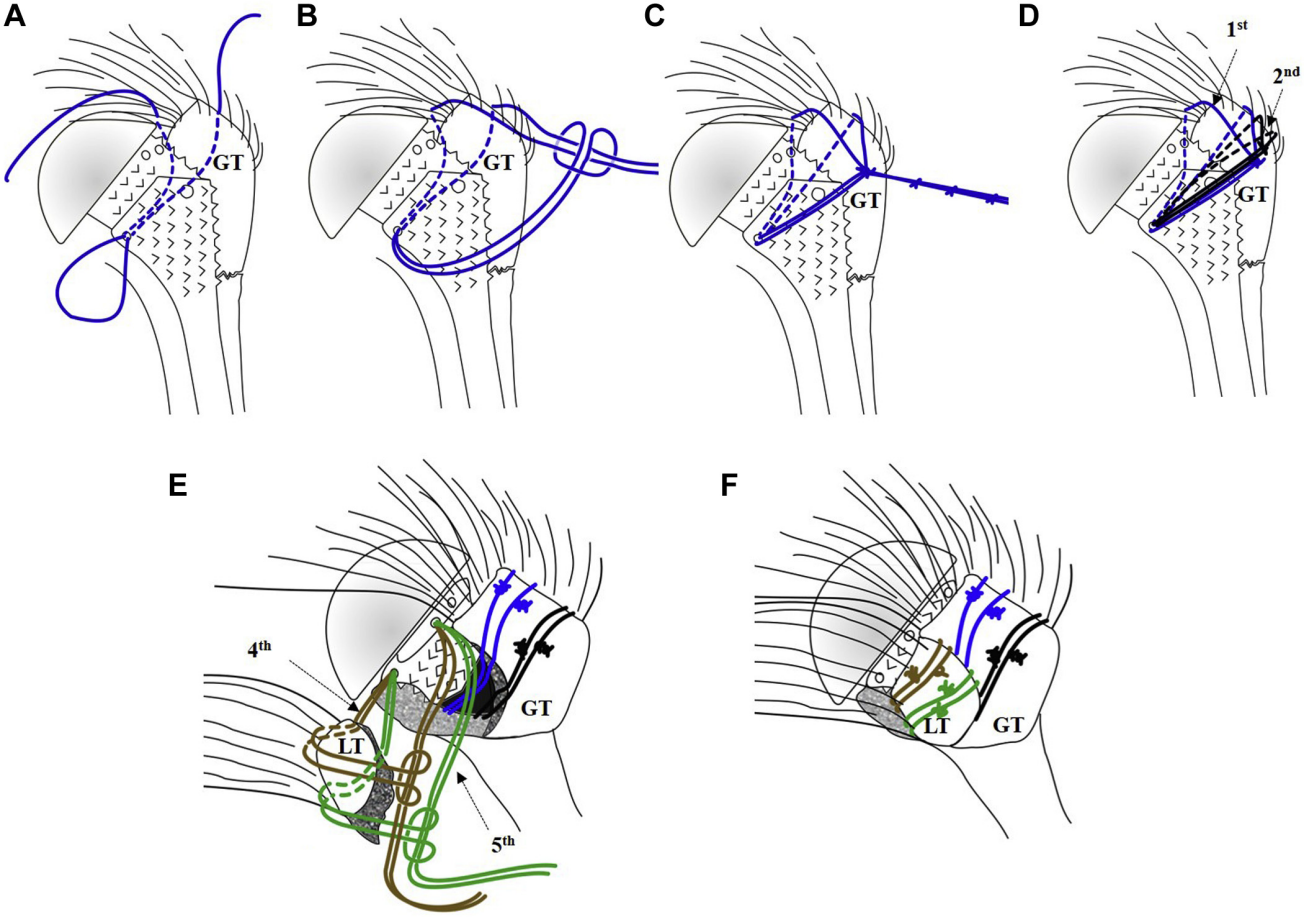
The greater and lesser tuberosity (GT and LT) reattachment with the Cow-Hitch technique using 4 Cow-Hitch cerclages. (**A**) The passing of the first loop. (**B**) The development of the double-loop cerclage with the free limbs. (**C**) Each cerclage is secured with several half hitches. (**D**) The Cow-Hitch cerclage for the GT reattachment. (**E**) The pathway of the 3^rd^ and 4^th^ double-loop cerclage with was used to reattach the LT. (**F**) The final reattachment of the GT and LT.

In total 4 sutures (MaxBraid sutures ♯5; ZimmerBiomet) were used to reattach the two tuberosities with the CH technique (Fig. 2).^7^^,^^12^

#### GT reattachment

The first stich was set at the supraspinatus tendon—GT transition stitching from outside into the intraarticular space. The created loop was passed through the medial prosthetic shaft hole (Fig. 2*A*). The major loop was then folded to develop two minor loops, which were positioned parallel to each other, so that the two free limbs could be passed through the double loop (Fig. 2*B*). Herewith a cerclage mechanism is created which allows developing tension on the suture by pulling on the free limbs. The self-locking mechanism of the cerclage prevents tension loss of the construct. No hemostats were necessary to secure the knots. The cerclage was secured with seven half hitches (Fig. 2*C*).

The second suture was used to create the exactly same double loop cerclage with the only difference being the suture was passed at the infraspinatus tendon-GT border which is more inferior (Fig. 2*D*; "1^st^ and 2^nd^"). After anatomical reduction of the GT, the humerus was held in 30° abduction and 20° of external rotation to tension and secure the cerclages.

#### LT reattachment

Two sutures were used to create two CH loops for the fixation of the LT. The sutures were passed through the subscapularis tendon-LT interphase. The created major loop was passed through the anterior hole of the prosthetic collar. With the major loop, two minor loops were created by folding the major loop. The free limbs were passed through the two minor loops to create the CH cerclage. The second CH was created in the exact identical way with the only difference that the sutures were passed more inferior through the subscapularis-bone interphase (Fig. 2*E*). After anatomical reduction of the LT, the CH were tensioned in 20° of internal rotation. Each CH cerclage was secured with 7 single knots (Fig. 2*F*).

The humerus with the implanted fracture prosthesis together with the rotator cuff tendons were then dissected free. The infraspinatus and subscapularis tendons were mounted each with two MaxBraid No. 5 sutures for the purpose of biomechanical testing. After cutting the shafts to an equal length, perpendicular to its axis 130 mm distal to the neck of the prosthesis, shafts were rigidly mounted in an aluminum cylinder being aligned perpendicular to the ground plate.

### Experiment apparatus

**Figure 3:**
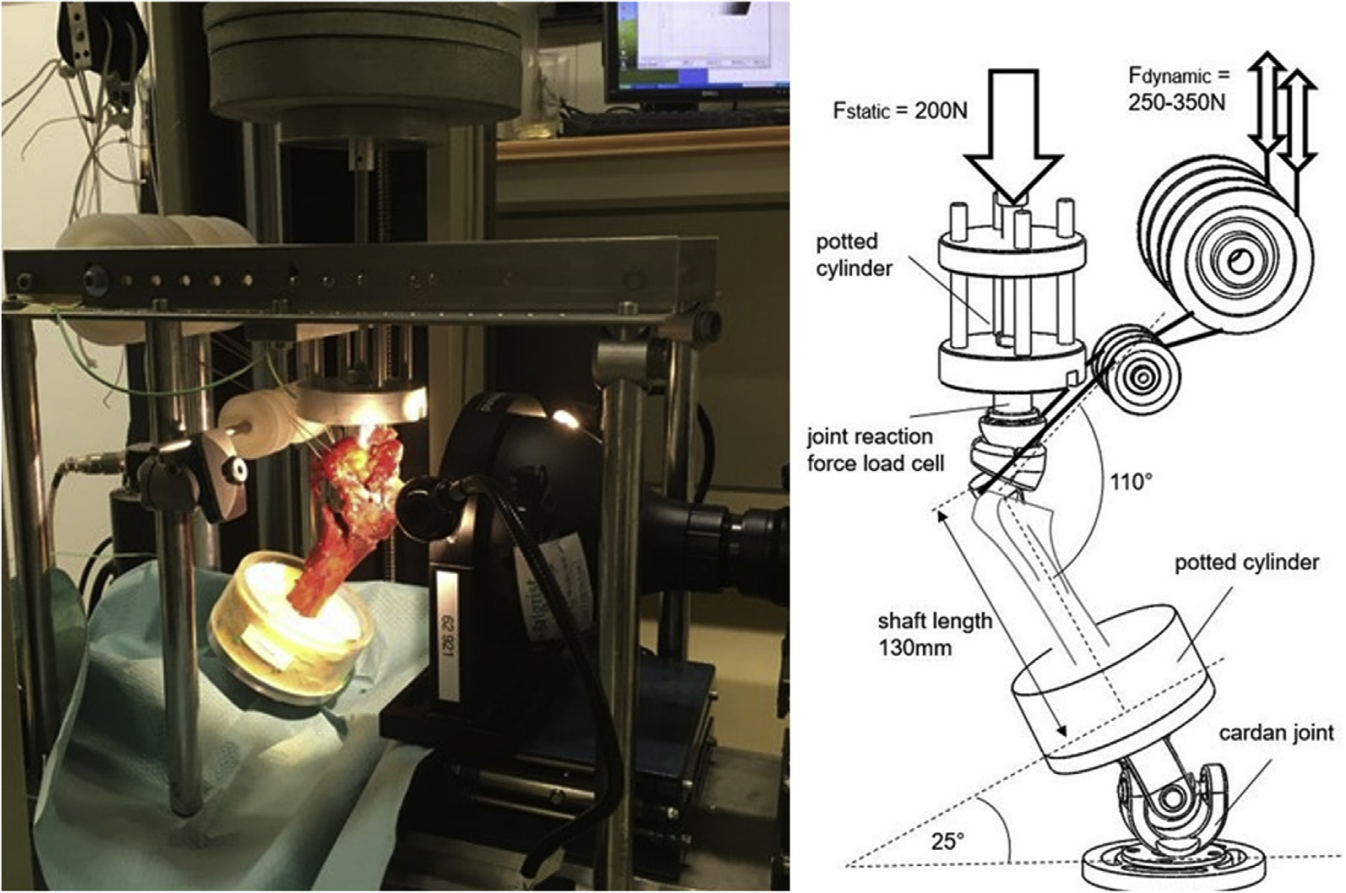
Schematic and photograph of the test setup.

Specimens were tested in a previously described setup^7^ using a universal material testing machine (Zwick 1456; Zwick GmbH, Ulm, Germany) (Fig. 3). A total of 5000 tension cycles of 250-350 N with 0.5 Hz were synchronically applied on these tendons via sutures, in line with the physiological line of action of the two respective muscles. Pullout testing was initiated with a preload of 50 N and a displacement rate of 0.5 mm/sec. Displacement and force were recorded and quantified as well as failure mode during testing or after pullout.

### Measurement of fracture displacement

The tuberosity displacement in the anteroposterior (AP), mediolateral (ML), and inferosuperior (IS) directions of each specimen was measured as the primary outcome parameters. To measure displacement of the bone fragments, a 5 degree of freedom coordinate measurement device was used (MicroScribe; Immersion Corp., San Jose CA, USA). Points were measured at 0, 10, 250, 500, 1000, 2000, 3000, 4000, and 5000 cycles.

### Statistical analysis

Normal distribution of data was assessed with the Shapiro-Wilk test. Descriptive data were calculated using mean and standard deviation (SD). The two groups were compared with the unpaired T-test (normal distribution) and with the Mann-Whitney U-test (non-normal data). Significance was set as *P* < .05, and all *P* values were two-tailed.

Data obtained in each group were analyzed statistically by F-tests to compare variances, followed by unpaired, two-tailed t-tests with the statistical software GraphPad Prism 7.03 (GraphPad Software, San Diego, CA, USA). Results are reported with means and whiskers showing the 95% confidence interval. A *P* value < 0.05 was considered to be statistically significant.

## Results

### GT displacement

**Figure 4:**
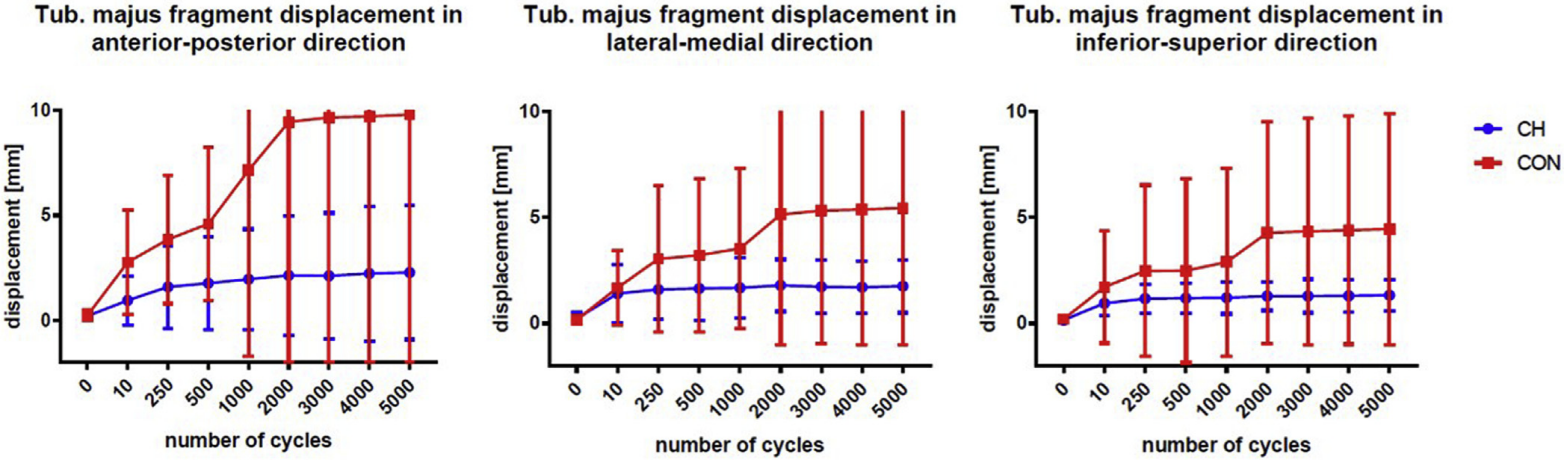
Fragment movement of the greater tuberosity (tuberculum majus, latin) relative to shaft during cyclic loading. Scatterplots with means and 95% confidence interval whiskers. Level of significance is defined as *P* < .05.

The initial GT fragment displacement during cyclic loading between the first and the tenth cycle showed a mean displacement in the AP direction of 0.9 ± 0.8 mm in the CH group and a mean displacement of 2.8 ± 2.2 mm (*P* = .136) in the control group. After 5000 loading cycles, the CH group showed a mean displacement distance of 2.3 ± 2.3 mm while the CON group showed a mean displacement of 9.8 ± 12.3 mm (*P* = .241) (Fig. 4*A* and Table I).

**Table I:** Summary of the measured displacement distances in mm of the greater and lesser tuberosity.

Displacement direction	CH GT displacement in mm			Conventional GT displacement in mm		
	AP	ML	IS	AP	ML	IS
10 cycles	0.9 ± 0.8	1.4 ± 1.0	0.9 ± 0.4	2.8 ± 2.2	1.7 ± 1.5	1.7 ± 2.3
5000 cycles	2.3 ± 2.3	1.8 ± 0.9	1.3 ± 0.5	9.8 ± 12.3	5.5 ± 5.6	4.5 ± 4.7
Displacement direction	CH LT displacement in mm			Conventional LT displacement in mm		
	AP	ML	IS	AP	ML	IS
10 cycles	0.4 ± 0.2	2.3 ± 0.9	0.9 ± 0.9	1.4 ± 0.8	1.8 ± 0.6	1.8 ± 0.9
5000 cycles	2.3 ± 3.3	7.2 ± 5.7	1.9 ± 1.2	4.0 ± 2.8	6.3 ± 3.6	3.1 ± 1.8

*CH*, Cow Hitch; *GT*, greater tuberosity; *AP*, anteroposterior; *ML*, mediolateral; *IS*, inferior-superior; *LT*, lesser tuberosity.

The mean fragment displacement in the ML direction was 1.4 ± 1.0 mm in the CH group and 1.7 ± 1.5 mm (*P* = .641) in the control group after the first 10 loading cycles and 1.8 ± 0.9mm and 5.5 ± 5.6mm (*P* = .159) in the CH control group, respectively, after 5000 cycles (see Fig. 4*B* and Table I).

In the IS direction, the mean displacement of the GT after the first 10 cycles was 0.9 ± 0.4mm in the CH group and 1.7 ± 2.3mm in the control group (*P* = .521). After 5000 cycles, the mean displacement of the CH group was 1.3 ± 0.5mm and 4.5 ± 4.7mm (*P* = .216) in the control group (Fig. 4*C* and Table I). The mean and SD of all displacement measurements of the CH group for the measurement in all three directions was 1.0 ± 0.6 mm, which is significantly smaller than that in the CON group (4.8 ± 3.6, *P* < .001).

### LT displacement

**Figure 5:**
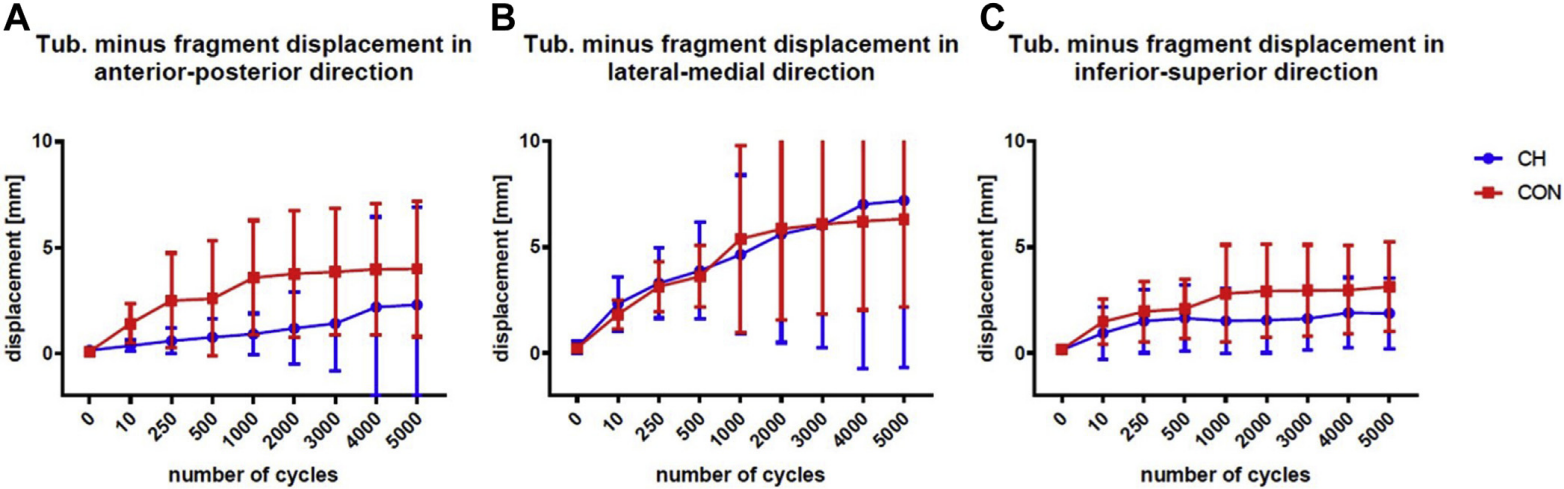
Fragment movement of lesser tuberosity (tuberculum minus, latin) relative to shaft during cyclic loading. Scatterplots with means and 95% CI whiskers. Level of significance is defined as *P* < .05.

The LT in the CH group showed a mean AP displacement of 0.4 ± 0.2mm and 1.4 ± 0.8mm in the control group (*P* = .087) after the initial 10 loading cycles. After 5000 cycles, the mean displacement in the CH group was 2.3 ± 3.3 and 4.0 ± 2.8mm in the control group (*P* = .359) (Fig. 5*A* and Table I).

The mean ML displacement of the LT in the CH group was 2.3 ± 0.9 and 1.8 ± 0.6 (*P* = .275) in the control group after the first ten loading cycles. After 5000 cycles, the mean displacement was 7.2 ± 5.7 in the CH group and 6.3 ± 3.6mm in the control group (*P* = .963) (Fig. 5*B* and Table I).

In the IS direction, the mean displacement of the LT fragment after the first ten cycles was 0.9 ± 0.9mm in the CH group and 1.8 ± 0.9mm in the control group (*P* = .413). After 5000 cycles, the mean displacement in the CH group was 1.9 ± 1.2mm and 3.1 ± 1.8mm in the CON group (*P* = .189) (Fig. 5*C* and Table I). The mean and SD of the LT displacement was 1.7 ± 1.5 in the CH group and 2.0 ± 1.1 in the control group (*P* = .170).

## Discussion

The aim of this study was to biomechanically compare the strength of the CH fixation technique for greater and LT reattachment to the CON technique, which is recommended by the manufacturer of the implant. Despite only two sutures being used for the CH fixation of the GT fragment, the displacement was smaller in all measured directions than that in the CON group in which four sutures were used. The absolute final displacement distances between the two groups showed significant differences in absolute values; however, the differences were not statistically significant. The main reason for the lack of statistical significance was the enormous scatter of the values in the CON group. The mean and SD values measured in the CH group (1.0 ± 0.6 mm) were substantially and significantly smaller than those in the CON group (4.8 ± 3.6mm, *P* < .001). This variability of fragment stability observed in the CON group—which was absent in the CH group—corresponds to the clinical reality, in which secondary tuberosity displacement is the most common complication after fracture HA which also results in variable outcomes.^2^^,^^4^^,^^10^ The narrow SD of GT displacement in the CH group reflects that very consistent and reproducible mechanical stabilizations can be achieved with this technique. Whether the improved mechanical stabilization of the tuberosity fragments results in a higher healing of the tuberosity fragments in vivo has to be verified in a clinical study.

The LT displacement in the AP and IS directions was lower in the CH group, surprisingly the displacement distance in the ML direction, which is biomechanically important for the subscapularis-LT unit, was higher in the CH group. It is possible that the "around-the-world" single-loop suture, which encircled the LT and GT fragments in the CON group, is a very efficient mechanical stabilizer. Maybe, the use of an "around-the-world" CH cerclage might have improved LT stabilization. In a further study, the feasibility and stability of an "around-the-world" CH cerclage for reattachment of the LT in fracture HA, but also for LT reattachment during total shoulder arthroplasty, will be tested.

In our view, the reason for malhealing or nonhealing of tuberosities in fracture HA depends on the fixation technique and restoration of the anatomy of the proximal humerus. Restoration of humeral anatomy was not investigated in this study, which is a limitation. Nevertheless, the prosthesis was implanted in a standardized manner in both groups to ensure comparability. In this study, the prosthesis was implanted in a standardized manner in both groups to guarantee comparability. For the stem implantation height, the upper edge of the pectoralis major tendon, at its attachment area, was taken as a reference. In both groups, the prosthesis was implanted in 20° retroversion. The head size was determined based on the head size from the original computed tomography. During the experiments, however, it was noted that certain shoulders would require individual adjustment of the implantation height and/or rotation to more precisely reproduce anatomy. We suspect that the stability of tuberosity fixation is higher in a shoulder in which the premorbid anatomy of the proximal humerus could be restored more accurately. The restoration of the proximal humerus anatomy after implantation of the HA was not investigated in this study, which is a limitation. Nevertheless, we explicitly ensured that implantation was standardized, which may be a confounding factor which is, however, compensated by randomization of the specimens and furthermore corresponds to a frequent clinical practice.

In addition to a standardized vs. an albeit clinically often not obtainable reconstruction, the following limitations apply:

- It is not known whether vital reactions such as resorption of the tuberosities influence the results. Therefore, it remains to be documented that the superior mechanical in-vitro stabilization results in higher tuberosity healing rates.
- The differences in the results were substantial but not statistically significant because of the enormous variability of the results in the CON group. We feel that precisely this variability, which was not present in the CH group, is a strong argument in favor of the CH technique, and increasing the number of specimens would not change this essential experimental observation.
- We did only compare two techniques. But the study documents reliability, reproducibility, and quantified stability measured in displacement provoked by cyclic loading and may, therefore, provide a benchmark for further studies.

Despite limitations, the study reproduced our clinical experience: Standard fixation of the tuberosities may or may not be associated with secondary fragment displacement and anatomical healing. Early clinical experience, which will be reported soon, at least suggests that anatomical tuberosity healing can be obtained more reliably with the CH technique. We feel that alternative techniques for tuberosity fixation should be compared to the CH technique as well experimentally as clinically.

## Conclusion

The reattachment of the GT in fracture HA with the use of two double-loop suture cerclages is reliably and reproducibly associated with minimal fragment displacement upon cyclical loading as opposed to the fixation with 4 sutures in a single-loop configuration, which yields highly variable stability. Further studies should compare tuberosity stability with the current results of CH fixation and focus on further improvements of LT fixation.

## Disclaimers

*Funding:* Zimmer Biomet GmbH, a company duly organized and existing under the laws of Switzerland with registered office at Sulzerallee 8, 8404 Winterthur, Switzerland (hereinafter “Zimmer Biomet”), provided the materials used for the study. No payments were made to an author or his family.

*Conflicts of interest:* C.G. received royalties and consultant payments from Zimmer Biomet, which is not related to the subject of this work. J.J.P.W. is a consultant for the Wright Medical Group, which is not related to the subject of this work. The other authors (F.G., E.B., L.E., P.B., K.W., S.B.) certify that he or she have no commercial associations (e.g., consultancies, stock ownership, equity interest, patent/licensing arrangements, and so forth) that might pose a conflict of interest in connection with the submitted article.
